# A retrospective real-world study of the current treatment pathways for myelofibrosis in the United Kingdom: the REALISM UK study

**DOI:** 10.1177/20406207221084487

**Published:** 2022-03-28

**Authors:** Adam J. Mead, Nauman M. Butt, Waseem Nagi, Alastair Whiteway, Suriya Kirkpatrick, Ciro Rinaldi, Catherine Roughley, Sam Ackroyd, Joanne Ewing, Pratap Neelakantan, Mamta Garg, David Tucker, John Murphy, Hitesh Patel, Rozinder Bains, Gavin Chiu, Joe Hickey, Claire Harrison, Tim C. P. Somervaille

**Affiliations:** NIHR Oxford Biomedical Research Centre and MRC Molecular Haematology Unit, MRC Weatherall Institute of Molecular Medicine, John Radcliffe Hospital, University of Oxford, Headington, Oxford OX3 9DS, UK; The Clatterbridge Cancer Centre NHS Foundation Trust, Liverpool, UK; Mid Essex Hospital Services NHS Trust, Chelmsford, UK; North Bristol NHS Trust, Bristol, UK; North Bristol NHS Trust, Bristol, UK; United Lincolnshire Hospitals NHS Trust, Boston, UK; East Kent Hospitals University NHS Foundation Trust, Canterbury, UK; Bradford Teaching Hospitals NHS Foundation Trust, Bradford, UK; University Hospitals Birmingham NHS Foundation Trust, Birmingham, UK; Royal Berkshire NHS Foundation Trust, Reading, UK; University Hospitals Leicester NHS Trust, Leicester, UK; Royal Cornwall Hospital NHS Trust, Truro, UK; University Hospital Monklands, NHS Lanarkshire, Airdrie, UK; Royal Albert Edward Infirmary, Wrightington, Wigan and Leigh NHS Foundation Trust, Wigan, UK; Novartis Pharmaceuticals UK Limited, Camberley, UK; Novartis Pharmaceuticals UK Limited, Camberley, UK; OPEN Health, Marlow, UK; Guy’s and St Thomas’ NHS Foundation Trust, London, UK; Cancer Research UK Manchester Institute, Manchester, UK; The Christie NHS Foundation Trust, Manchester, UK

**Keywords:** duration of therapy, myelofibrosis, myeloproliferative neoplasms, real-world data, ruxolitinib

## Abstract

**Background:**

Myelofibrosis (MF) is a blood cancer associated with splenomegaly, blood count abnormalities, reduced life expectancy and high prevalence of disease-associated symptoms. Current treatment options for MF are diverse, with limited data on management strategies in real-world practice in the United Kingdom.

**Methods:**

The REALISM UK study was a multi-center, retrospective, non-interventional study, which documented the early management of patients with MF. The primary endpoint was the time from diagnosis to active treatment.

**Discussion:**

Two hundred patients were included (63% [*n* = 126/200] with primary MF; 37% [*n* = 74/200] with secondary MF). Symptoms and prognostic scores at diagnosis were poorly documented, with infrequent use of patient reported outcome measures. ‘Watch and wait’ was the first management strategy for 53.5% (*n* = 107/200) of patients, while the most commonly used active treatments were hydroxycarbamide and ruxolitinib. Only 5% of patients proceeded to allogeneic transplant. The median (IQR) time to first active treatment was 46 days (0–350); patients with higher risk disease were prescribed active treatment sooner.

**Conclusion:**

These results provide insight into real-world clinical practice for patients with MF in the United Kingdom. Despite the known high prevalence of disease-associated symptoms in MF, symptoms were poorly documented. Most patients were initially observed or received hydroxycarbamide, and ruxolitinib was used as first-line management strategy in only a minority of patients.

**Plain Language Summary:**

**Background:** Myelofibrosis is a rare blood cancer associated with symptoms that can seriously affect a patient’s daily life, such as enlarged spleen and decreased white and red blood cells. Although several treatments are available for patients with myelofibrosis, it is not clear which ones clinicians use most frequently.

**Methods:** We aimed to review which treatments are usually given to patients with myelofibrosis in the UK, by collecting information from the medical records of 200 patients with myelofibrosis treated in different centres across the UK.

**Results:** The results showed that the symptoms patients experienced were not always written down in the medical records. Similarly, clinical scores based on patient characteristics (which clinicians use to try to predict if a patient will respond to treatment well or not) were also missing from the medical records. Clinicians also rarely asked patients to complete questionnaires that try to measure the impact of myelofibrosis and its treatment on their health. The most common approach for patients with myelofibrosis in the UK was ‘watch and wait’, which over half of patients received. The most common drugs used for treatment were hydroxycarbamide and ruxolitinib; only a very small proportion of patients received a bone marrow transplant. On average, patients waited for 46 days before receiving a treatment, although patients considered to have a more aggressive type of disease received treatment sooner.

**Conclusion:** The results of this study suggest that medical records can be missing key information, which is needed to decide which is the best way to treat a patient with myelofibrosis. They also suggest that clinicians in the UK prefer observation to treatment for a large number of patients with myelofibrosis. This could mean that the approach used for many patients with myelofibrosis does not help them to control symptoms that have an impact on their daily lives.

## Introduction

Myelofibrosis (MF) is a myeloproliferative neoplasm (MPN) that presents as either a primary disorder (primary myelofibrosis [PMF]) or secondary to polycythemia vera (PV) or essential thrombocythemia (ET).^1–3^ MPNs are rare disorders, with reported annual incidence rates ranging from 0.01 to 2.61, 0.21 to 2.27, and 0.22 to 0.99 per 100,000 for PV, ET, and PMF, respectively.^4^

The clinical presentation of PMF is highly heterogeneous, with variable splenomegaly, blood count abnormalities, reduced life expectancy and high prevalence of diverse, disease-associated symptoms with consequently reduced quality of life.^5,6^ Aside from the presentation heterogeneity, estimating patient prognosis can be difficult. The International Prognostic Scoring System (IPSS) identifies features associated with poor outcome of PMF and can be used to estimate prognosis at the time of diagnosis, whereas the Dynamic International Prognostic Scoring System (DIPSS) can be used to inform prognosis at any time during the course of the disease.^7,8^ Both the IPSS and DIPSS use a series of factors that are independently associated with poor outcome, including age  > 65 years, hemoglobin level  < 100 g/L, leukocyte count  > 25 × 10^9^/L, circulating blasts ⩾ 1% and the presence of constitutional symptoms (fever, weight loss, and night sweats), with the DIPSS plus score incorporating thrombocytopenia, red blood cell (RBC) transfusion dependency and unfavorable karyotype as additional prognostic factors. Based on the presence of these factors, patients can be stratified according to their IPSS, DIPSS or DIPSS plus score into four risk categories: low, intermediate-1, intermediate- 2 and high-risk.^7,8^ Key clinical management decisions are informed by this risk stratification,^9^ but in standard clinical care it remains unclear which prognostic score is used and how often this risk stratification is documented.

Current treatment options for MF (e.g. JAK inhibitors, hydroxycarbamide, thalidomide) typically manage MF-related symptoms, but are not considered disease modifying; asymptomatic and low-risk patients are often observed without active treatment (the ‘watch and wait’ management strategy).^3^ The only curative treatment option is allogeneic hematopoietic stem cell transplantation (HSCT); however, this approach is associated with high rates of mortality and morbidity, particularly in patients over 45 years of age.^1,3^

Analysis of real-world data recorded in the Hematological Malignancy Research Network showed that patients with MF had the poorest prognosis of all patients with MPNs.^10^ Due to the multitude of available management options and the lack of clear differences in prognosis between strategies, treatment choices can be heterogeneous and real-world data on the treatment pathways of patients with MF in the United Kingdom are limited.^5,11^ For example, data are lacking on the proportion of patients managed with the ‘watch and wait’ strategy; given that effective therapies are available to treat symptoms, it is important to know whether patients that are in ‘watch and wait’ may benefit from active treatment instead.

The availability of new reduced-intensity, non-myeloablative conditioning strategies has increased transplant frequency in patients with MF,^12^ but it still remains a high-risk approach for the vast majority of patients; real-world data on the number of patients that receive allogeneic HSCT is needed. Information on the durability of treatment, survival rates and causes of death in the real world is also important to guide clinical decisions. Finally, the consistency of documentation of disease features needs to be assessed, as it is vital for effective and continuous patient care within multidisciplinary healthcare teams.

The REALISM UK retrospective observational study used real-world data to document the early management strategies for patients with MF in routine clinical practice across the United Kingdom. Other aims included a description of patient demographics and clinical characteristics at the time of diagnosis with MF, as well as the type and duration of chosen management strategies.

## Materials and methods

### Study design and participants

REALISM UK was a multi-center, retrospective, non-interventional study carried out in 15 National Health Service (NHS) hospitals (secondary and tertiary care centers) across the United Kingdom. The centers participating in the study were the Churchill Hospital, Oxford; Royal Berkshire Hospital, Reading; Guy’s Hospital, London; University Hospital Monklands, Airdrie; The Clatterbridge Cancer Center, Liverpool; The Christie NHS Foundation Trust, Manchester; Southmead Hospital, Bristol; Broomfield Hospital, Chelmsford; Bradford Royal Infirmary, Bradford; Birmingham Heartlands Hospital, Birmingham; Pilgrim Hospital, Boston; Kent and Canterbury Hospital, Canterbury; Royal Albert Edward Infirmary, Wigan; Leicester Royal Infirmary, Leicester; and Torbay and South Devon Hospital, Torbay. Each center recruited up to a maximum of 25 patients. Data collection started on January 2018 and finished in January 2019.

Patients who gave their consent to participate in the study (applicable to living patients only) were eligible if aged ⩾ 18 years at the time of MF diagnosis and had at least one follow-up visit following diagnosis. Patients diagnosed with MF 5 years prior to the cut-off date for the study observation period/data collection (31 January 2019) were included in the study, whereas patients diagnosed less than 6 months before data collection were excluded. Patients were also excluded from the study if their medical records were unavailable for review.

Eligible patients were first numbered in consecutive order according to their date of diagnosis of myelofibrosis and a study-specific random number generator was used by the centers to choose the study sample from this list. The chosen sample of patients was approached for consent and the process was repeated with the remaining eligible patients if enough consenting patients were not selected in the first round of random sample selection.

The duration of the study observation period extended from the date of diagnosis of MF to the date of data collection. Data on patient demographics, clinical characteristics, MF management strategies and outcomes were collected from patients’ available medical records, which mainly consisted of physical notes and computer records where access was available. The data were collected at some sites by the external study team and at other sites by the local research team.

This study received a favorable opinion from the East Midlands–Leicester South Research Ethics Committee (REC reference number: 17/EM/0425). Appropriate permissions from the Research and Development (R&D) departments of each participating NHS trust/health board were also obtained. The study was conducted in accordance with applicable legal and regulatory requirements and research practice guidelines. The reporting of this study conforms to the STROBE statement.^13^

### Endpoints

The primary endpoint of the study was the time period elapsing between initial diagnosis of MF and commencement of first active intervention for MF (i.e. the time that a patient is initially managed by a ‘watch and wait’ approach or time to first treatment [TTFT]). A key secondary endpoint of the study was the distribution of patient characteristics at the time of MF diagnosis, including demographics (age and sex), diagnosis method (and criteria used), distribution of MF types (primary/post-ET/post-PV), mutational status, MF symptoms, spleen size, IPSS prognostic score (or alternative prognostic score if IPSS was not documented), and percentage of patients with IPSS documented at diagnosis. Other secondary endpoints included the frequency of anemia, thrombocytopenia, and serious infections.

For the purposes of this study, ‘watch and wait’ was defined as a period of time in patient management where patients were not receiving active treatment for MF. Active treatment was defined as any pharmacological intervention to treat the disease, and included targeted therapies (such as JAK inhibitors), antineoplastics (hydroxycarbamide, busulphan, interferon-α), miscellaneous agents (anagrelide, azacitidine, thalidomide), and allogeneic HSCT. Other treatments such as erythropoietin or blood transfusion were considered to be supportive care.

### Statistical analysis

Analyses were descriptive in nature. Distributions and descriptive statistics of both central tendency (medians and arithmetic or geometric means) and dispersion (standard deviation [SD], interquartile range [IQR]) were presented for quantitative variables. Nominal variables were described with frequencies and percentages, whereas ordinal variables were described using medians and IQRs.

The primary endpoint of time between initial diagnosis of MF and commencement of first active treatment for MF (i.e. time that the patient was initially managed by ‘watch and wait’ strategy) was described alongside proportions of patients that received an active treatment during the study observation period. For patients who received allogeneic HSCT as their first active treatment, the period of ‘watch and wait’ was calculated from the date of diagnosis to the date of decision to proceed with allogeneic HSCT.

The Kaplan-Meier method was used to analyze time from diagnosis of MF to initiation of first active treatment. Patients who did not commence active treatment during the study observation period were censored at the last observed date of follow-up.

As clinical circumstances and the number of available treatments at the time may be a factor affecting the timing at which a first active intervention was selected, a Cox regression analysis was performed to determine whether there was a statistically significant relationship between date of diagnosis and duration of initial management by ‘watch and wait’. All analyses were performed in Microsoft Excel and STATA (version 14).

## Results

### Patient demographics and disease characteristics

In total, 200 patients were enrolled in the study. Patient demographics are summarized in Table 1. The median patient age was 69.7 years, and 40.5% (*n* = 81) of the patients were female. Out of all eligible patients, 63% (*n* = 126/200) were diagnosed with PMF and 37% (*n* = 74/200) with secondary MF; 55.4% (*n* = 41/74) of secondary MF patients were post-PV and 44.6% (*n* = 33/74) were post-ET.

**Table 1.** Patient demographics and disease characteristics.

Demographics^a^	Patients (*n* = 200)
Females	81 (40.5%)
Median (IQR) age (years)	69.7 (63.5–75.7)
Age range (years)	20.3 to 91.8
Disease characteristics	
MF diagnosis	
Primary MF	126 (63%)
Secondary MF (post-ET)	33 (16.5%)
Secondary MF (post-PV)	41 (20.5%)
*JAK2*^V617F^ mutation	
Mutational testing performed	174 (87%)
Positive^b^	141 (81%)
Negative^b^	33 (19%)
Data not available	26 (13%)
Calculated IPSS risk category^c^	
Low – 0	15 (7.5%)
Intermediate – 1	58 (29%)
Intermediate – 2	59 (29.5%)
High ⩾ 3	39 (19.5%)
Scoring items unavailable	29 (14.5%)

ET, essential thrombocythemia; IPSS, International Prognostic Scoring System; IQR, interquartile range; JAK, Janus Kinase; MF, myelofibrosis; PV, polycythemia vera.

a Data are expressed as *n* (%) unless otherwise indicated.

b Percentage expressed as the proportion of patients for whom *JAK2*^V617F^ mutational testing was performed (*n* = 174).

c IPSS scores were calculated from recorded scoring items when available. Multiple scoring items were unavailable for 5 patients, but the recorded information on the remaining items was enough to place them in the high-risk category.

Across the cohort of 200 patients, 87% (*n* = 174) were tested for the *JAK2*^V617F^ mutation, whereas 30% (*n* = 60) were tested for *CALR* exon 9 insertion/deletion mutations, and only 14.5% (*n* = 29) were tested for *MPL*^W515^ mutations: this is consistent with sequential testing in patients (i.e. most patients were only tested for other mutations if the *JAK2*^V617F^ mutation was not detected). The *JAK2*^V617F^ mutation was detected in 81% of tested patients (*n* = 141/174), whereas *CALR* exon 9 insertion/deletion and *MPL*^W515^ mutations were detected in 35 and 11 patients, respectively. It should be noted that more than one mutation was recorded for some patients. Out of 200 patients, 18 were noted as having no mutations detected and 5 patients were recorded as not having been tested for mutations, while mutation status was recorded as unknown for another 7 patients.

Prognostic scoring was poorly documented in assessed patient records. Scores were recorded in 11 instances for IPSS, 12 for DIPSS, 10 for DIPSS plus, and 3 for other scoring systems; 3 patients had more than one type of score recorded. Prognostic scores were not recorded in the clinical notes for 168 patients. IPSS prognostic scores could be calculated where appropriate data from medical records on scoring items (i.e. age, hemoglobin level, leukocyte count, presence of circulating blast cells, and constitutional symptoms) were available; however, at least one scoring item was unavailable for 14.5% (*n* = 29) of patients, with percentage of circulating blasts being the most common missing item.

At diagnosis, 49% (*n* = 98/200) of patients had IPSS categories of intermediate-2 (Int-2) or high-risk. As the scores herein were calculated assuming a score of 0 where data were unavailable, they may underestimate the true profile of IPSS scores in this cohort. Multiple scoring items were unavailable for 5 patients, but the recorded information on the remaining items was enough to place them in the high-risk category.

Overall, 58.5% of patients (*n* = 117) presented with symptoms which prompted investigation for and diagnosis of MPN. Only twenty out of all patients had a documented MPN Symptom Assessment Form Total Symptom Score (MPN-SAF TSS) during the observation period, highlighting how infrequently this is implemented in routine practice. The majority of patients were diagnosed on the basis of both patient-reported symptoms and laboratory tests. Signs and symptoms documented in patient records, as well as laboratory values collected up to 4 weeks prior to MF diagnosis, are summarized in Table 2.

**Table 2.** Signs, symptoms and laboratory values.

Signs and laboratory values recorded in patient notes at diagnosis	Patients (*n* = 200)
Splenomegaly	94 (47%)
Anemia	88 (44%)
Leucocytosis	7 (3.5%)
Thrombocytopenia	5 (2.5%)
Thrombocytosis	4 (2%)
Unexplained fever	2 (1%)
Laboratory values^a^	
Anemia (Hb < 10 g/dL)	63 (33.0%)
Thrombocytopenia (platelet count < 150x10^9^/L)	35 (18.3%)
Symptoms recorded in patient notes at diagnosis	
Unexplained tiredness	54 (27%)
Unintended weight loss	42 (21%)
Excessive sweating (especially at night)	31 (16%)
Shortness of breath	18 (9%)
Bone and joint pain	16 (8%)
Feeling of discomfort or abdominal pain	14 (7%)
Fullness, indigestion and a loss of appetite	14 (7%)
Pruritus	12 (6%)
Weakness	11 (6%)
Bleeding problems	6 (3%)
Palpitations	1 (1%)
Abdominal discomfort from enlarged liver	1 (1%)
Number of constitutional symptoms recorded in patient notes at diagnosis	
0	114 (57%)
1	45 (23%)
2	15 (8%)
None recorded^b^	26 (13%)

Hb, hemoglobin.

a Most recent results in 4 weeks prior to MF diagnosis (*n* = 191 patients with evaluable data).

b Some symptoms were recorded, but no constitutional symptoms.

Splenomegaly and anemia were the most common features of disease, recorded at diagnosis as present in 47% (*n* = 94) and 44% (*n* = 88) of all patients, respectively. However, the proportion of patients with anemia (defined as hemoglobin  < 10 g/dL) was lower when considering the most recent hemoglobin results in the 4 weeks prior to diagnosis of MF (63/191 patients, 33.0%). Other symptoms varied in frequency, with unexplained tiredness (27%, *n* = 54), unintended weight loss (21%, *n* = 42), and abnormal sweats (15.5%, *n* = 31) among the most commonly documented. No constitutional symptoms were recorded in patient notes from 57% of recruited patients (*n* = 114). It should be noted that the aforementioned signs and symptoms are those documented in the patient records, and therefore limited by the extent to which these were recorded by clinicians.

Overall, 70 patients (35%) required transfusions during the study observation period. Of these patients, 25/66 (37.8%) were recorded as intermediate-1 IPSS risk group at diagnosis, 13/66 (19.7%) were in the intermediate-2 group, and 6/66 (9.1%) were in the high-risk group. Throughout the observation period the IPSS risk group could have changed from that at diagnosis, which explains why some patients recorded as lower risk group at diagnosis required transfusions. Although anemia was common, only a small number of patients received supportive therapies for anemia during the 6-month period after initiation of a new core management strategy, with 9 patients receiving erythropoietin and 1 patient receiving danazol. As supportive therapies were not mutually exclusive, any single patient could have received more than one.

Thrombocytopenia was specifically documented in only five patients at diagnosis (2.5%); however, the most recent platelet count results in the 4 weeks prior to diagnosis of MF, showed thrombocytopenia (defined as platelet count  < 150 x 10^9^/L) in 35/191 patients (18.3%). There were 81 platelet transfusions recorded during the study observation period across 6 patients: 2 patients had the majority of these (1 was on ruxolitinib, the other on ruxolitinib and a JAK inhibitor clinical trial), while the remaining 4 patients only had 1 each (2 were on ruxolitinib and the other 2 on ‘watch and wait’); neither of these patients had received intensive chemotherapy or allogeneic HSCT. No patients were recorded to have received tranexamic acid.

Thirty-one patients received prophylactic therapies for infections during the 6-month period after initiation of core management strategies; description of prophylactic therapies for patients on ruxolitinib shows that over half of these patients received acyclovir during the observation period (Supplementary Table 1). Infections were recorded in 28 patients after initiation of various management strategies for MF; patients were treated with antibiotics, with amoxicillin, clarithromycin and meropenem being the most commonly used (in 19%, 17%, and 11% of these treatments, respectively).

### Time to first active treatment

**Figure 1.**
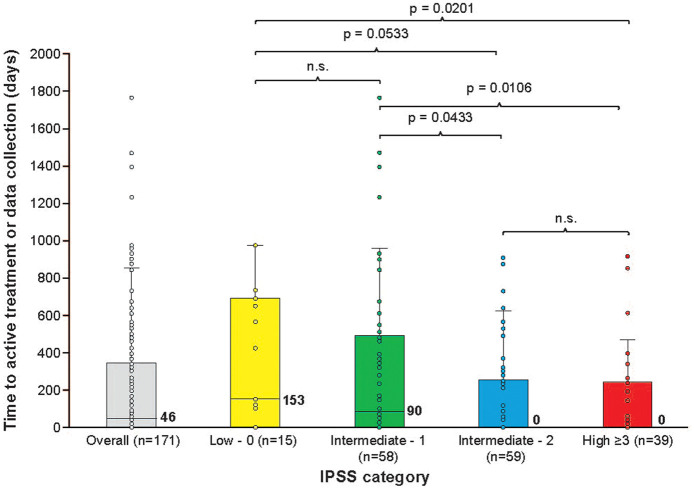
Median time to initiation of active treatment according to IPSS category. In the box plots, the boundary of the box closest to zero indicates the 25th percentile, a black line within the box marks the median, and the boundary of the box farthest from zero indicates the 75th percentile; median values are also shown to the right of each box. Whiskers above and below the box indicate the maximum and minimum range values, respectively. Points indicate individual patient values. Significance between groups was estimated by Cox regressions. IPSS, International Prognostic Scoring System; *n*.s., not significant.

Active treatment as defined in the Materials and Methods section was started at diagnosis for 46.5% (*n* = 93) of patients, with the remaining 53.5% (*n* = 107) on a period of ‘watch and wait’ as the initial core management strategy. Analysis of the median time (IQR) from diagnosis of MF to first active treatment (defined as any pharmacological intervention to treat MF) in the study population with available/calculable IPSS score at diagnosis (*n* = 171) at the time of data collection was 46 days (0–342), but showed significant heterogeneity across IPSS risk groups (Figure 1). The median time (IQR) to active treatment stratified by IPSS score was 153 days (0–667) in the low risk group, 89.5 days (0–473) in the intermediate-1 risk group, 0 days (0–251) in the intermediate-2 risk group and 0 days (0–216) in the high-risk group. Although patients with higher risk disease were prescribed active treatment sooner, a proportion of patients with symptomatic and/or high-risk disease were managed on a ‘watch and wait’ strategy, sometimes for prolonged periods (Figure 1).

Choice of first management strategy differed according to disease risk (Table 3). Patients were switched from a ‘watch and wait’ strategy to active treatment as risk score increased, and the percentage of patients treated with ruxolitinib also increased with risk score. There was no clear trend for hydroxycarbamide management according to IPSS risk level. Interferon-α and anagrelide were rarely used.

**Table 3.** Choice of first management strategy by IPSS group.^a^.

First management strategy	Low (0)	Intermediate – 1	Intermediate – 2	High > = 3
	Patients (*n* = 15)	Patients (*n* = 58)	Patients (*n* = 59)	Patients (*n* = 39)
Anagrelide	0 (0%)	1 (2%)	1 (2%)	1 (3%)
Clinical trial- other JAK-I	0 (0%)	0 (0%)	2 (3%)	0 (0%)
Hydroxycarbamide + anagrelide	0 (0%)	1 (2%)	2 (3%)	0 (0%)
Hydroxycarbamide	4 (27%)	10 (17%)	11 (19%)	10 (26%)
Interferon-α	0 (0%)	2 (3%)	1 (2%)	0 (0%)
Ruxolitinib	1 (7%)	9 (16%)	13 (22%)	10 (26%)
Watch and wait	10 (67%)	35 (60%)	29 (49%)	18 (46%)

IPSS, International Prognostic Scoring System; JAK-I, Janus Kinase inhibitor.

a Management strategies for patients for whom IPSS scoring was not available (*n* = 29) are not shown in this table.

### Type and duration of management strategies

The distribution of management strategies over the duration of the study observation period is shown in Supplementary Table 2. Management strategies were considered interventions to treat the disease, and included targeted therapies (such as JAK inhibitors), antineoplastics (hydroxycarbamide, busulphan, interferon-α), miscellaneous agents (anagrelide, azacitidine, thalidomide), and allogeneic HSCT, as well as ‘watch and wait’. All other therapies were considered supportive.

A summary of the number of courses prescribed of each of the most common management strategies – including those that persisted for 6 months or longer – documented in patient records is shown in Table 4. Overall, during the course of the study observation period, several different management strategies were used in patients diagnosed with MF. The most commonly used core strategies were ‘watch and wait’ (*n* = 134), ruxolitinib (*n* = 111) and hydroxycarbamide treatment (*n* = 68); patients could be recorded as managed by more than one treatment strategy at any given time. Overall, 107 patients were on a period of ‘watch and wait’ as first management strategy and 27 patients were on ‘watch and wait’ following active treatment.

**Table 4.** Documented MF management strategies.

Management strategy^a^	*n* (courses)	*n* (persisting ⩾ 6 months)
Watch and wait	134	81
Ruxolitinib	111	81
Hydroxycarbamide	68	44
Allogeneic HSCT follow-up^b^	10	5
Interferon-α	10	7
Ruxolitinib + hydroxycarbamide	9	7
JAK inhibitor (part of clinical trial)	8	4

HSCT, hematopoietic stem cell transplant; JAK, Janus Kinase; MF, myelofibrosis.

a Patients may have had more than one management strategy.

b Described as such in patient records.

In total, only 5% (*n* = 10/200) of patients were recorded as having undergone allogeneic HSCT during the study observation period. Of 39 patients who were aged < 70 at diagnosis, had IPSS risk score available at diagnosis and were in the intermediate-2 or high-risk groups, 3 patients (8%) underwent allogeneic HSCT, a slightly higher proportion than for the overall population. The mean age (SD) for patients receiving allogeneic transplant was 59.5 years old (6.3), ranging from 50.9 to 67.4 years old. No transfusions were recorded in the 8 weeks prior to MF diagnosis for any of these patients. In terms of previous management strategies, two patients had been in ‘watch and wait’ prior to undergoing allogeneic HSCT and the remaining eight had at least one active treatment. As shown by patient records, allogeneic HSCT was not used as first management strategy.

**Figure 2.**
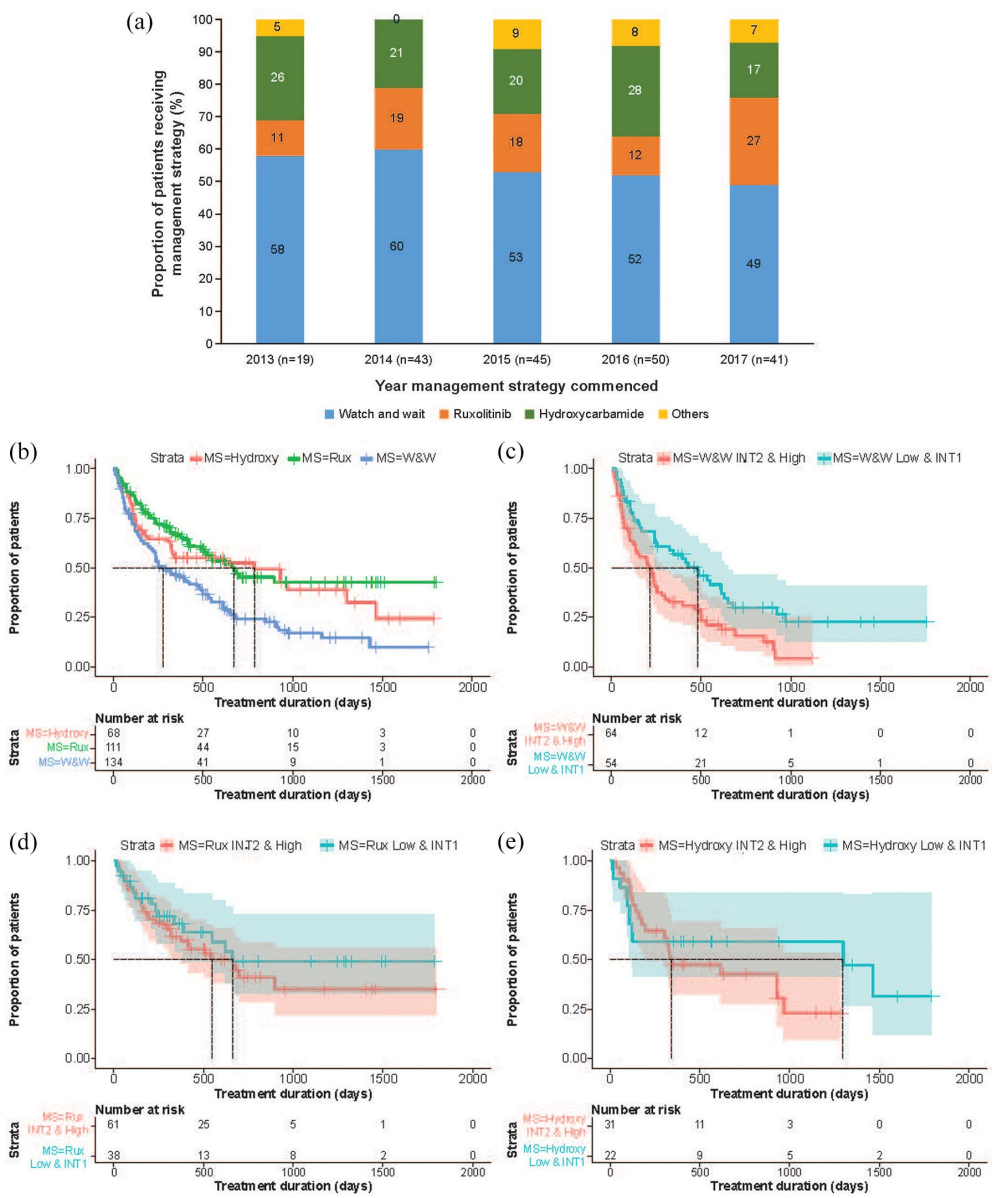
(a) Distribution of management strategies during study observation period by year of diagnosis. Represented are the percentages of patients managed with a certain strategy for each year. (b) Duration of the most common management strategies. Kaplan-Meier analysis of treatment duration was performed with patients censored at the time of initiation of first active treatment. (c) Duration of ‘watch and wait’ according to risk category. (d) Duration of ruxolitinib treatment according to risk category. (e) Duration of hydroxycarbamide treatment according to risk category. Shaded areas represent 95% CI. Hydroxy, hydroxycarbamide; INT1, intermediate-1; INT2; intermediate-2; MS, management strategy; Rux, ruxolitinib; W&W, watch and wait. ^a^One patient was recruited in 2018, and was treated with hydroxycarbamide.

The distribution of management strategies varied according to the year of diagnosis (Figure 2(a)), with a trend toward a decreased number of patients being managed on a ‘watch and wait’ strategy in later years. A similar trend was observed when assessing the time to initiation of first active treatment according to year of diagnosis (Table 5): median TTFT decreased over the years, reflecting an earlier start of active treatment. However, no clear trend emerged when analyzing first management strategy according to year of treatment initiation (Supplementary Table 3) and/or IPSS score (Supplementary Table 4).

**Table 5.** Time to initiation of first active treatment by year of diagnosis.

Time to initiation of first active treatment^a^	Year of MF diagnosis					
	2013	2014	2015	2016	2017	2018
*n* (patients)	20	43	46	50	40	1
Mean (days)	342.6	315.9	286.0	162.8	108.4	N/A
SD	516.7	421.1	372.0	229.8	160.4	
Median (days)	73.0	153.0	49.5	42.5	0.0	
IQR	0.0 to 620.3	0.0 to 521.0	0.0 to 522.8	0.0 to 321.3	0.0 to 203.3	
Range	0.0 to 1755.0	0.0 to 1463.0	0.0 to 1235.0	0.0 to 732.0	0.0 to 499.0	

IQR, interquartile range; MF, myelofibrosis; N/A, not applicable; SD, standard deviation.

a Including patients on ‘watch and wait’ at data collection.

During the study observation period, 40.5% (*n* = 45/111) of patients discontinued ruxolitinib treatment and 55.9% (*n* = 38/68) of patients discontinued hydroxycarbamide treatment. Figure 2(b) shows the duration of the most common management strategies for those patients that underwent them (*n* = 134 for ‘watch and wait’, *n* = 111 for ruxolitinib, and *n* = 68 for hydroxycarbamide). The median duration (IQR) of management strategies during the study observation period was shortest for ruxolitinib (541.5 days [313.5 to 998.8]), followed by hydroxycarbamide (608.0 days [407.5 to 988.5]), and ‘watch and wait’ (619.0 days [392 to 973.0]). Figure 2(c)–(e) show that treatment duration was longer for patients in the low and intermediate-1 risk categories across all three management strategies; duration of ‘watch and wait’ was significantly shorter for patients with higher risk disease (*p* = 0.0044, Wilcoxon rank-sum test; *p* = 0.0013, independent *t*-test). The duration of ruxolitinib treatment was significantly longer when used as a first management strategy (*p* = 0.047) (Supplementary Figure 1).

### Causes of death

**Figure 3.**
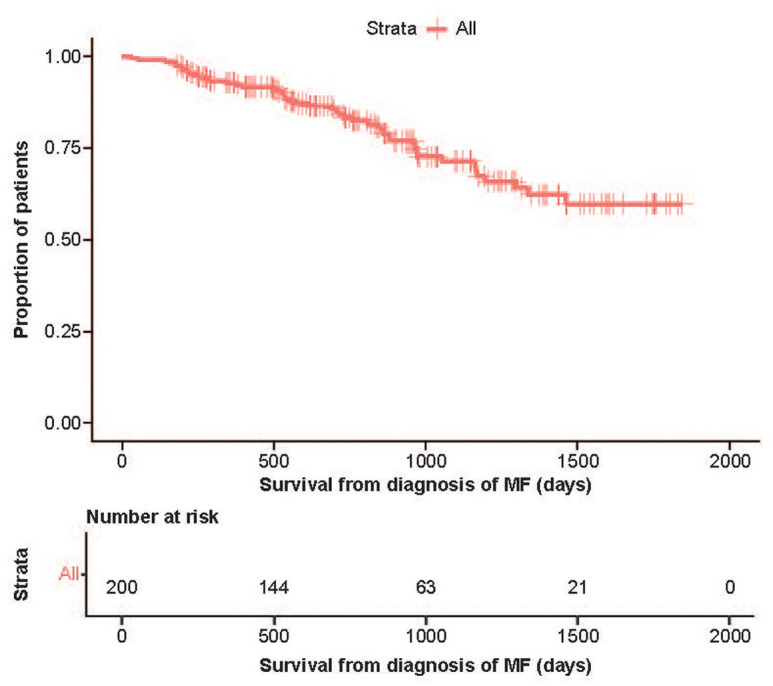
Kaplan-Meier survival analysis from time of diagnosis to end of observation period. MF, myelofibrosis.

Forty-seven deaths were recorded during the study observation period (Table 6). Kaplan-Meier survival analysis from time of diagnosis to the end of the observation period is shown in Figure 3. The cause of death was not available for 8 patients. Among patients with known death causes, 63.8% (*n* = 30) were MF-related, 12.8% related to other malignancies (including lung cancer and sigmoid colon cancer) and 6.4% to heart failure. Regarding clinical disease progression, several different types were assessed; the most common were progression to a more severe form of MF or progression to acute myeloid leukemia.

**Table 6.** Causes of death.

Category	*n* (patients)	% (*n* = 47)
Disease-related^a^	15	31.9%
Disease progression	10	21.3%
Infection	4	8.5%
Bleeding	1	2.1%
**Total MF-related causes**	**30**	**63.8%**
Other malignancy^b^	6	12.8%
Heart failure	3	6.4%
Not available	8	17%

MF, myelofibrosis.

a Recorded as disease-related with no full detail available.

b Other malignancies documented in patient records include lung cancer (4 patients), metastatic sigmoid colon cancer (1 patient), and tumor lysis syndrome of unknown origin (1 patient).

## Discussion

This real-world, observational retrospective study aimed to provide insight into current treatment pathways for patients with MF in the United Kingdom.

The most striking finding of our study was that ‘watch and wait’ was the most commonly used management strategy, even in patients with intermediate-2 or high-risk IPSS groups. The need to wait for multidisciplinary team decisions (as per NICE guidelines) before instituting treatments, as well as pending trial availability, could explain why some patients are initially managed with the ‘watch and wait’ strategy. Our results also show that hydroxycarbamide is still very frequently used in MF despite its effectiveness being lower than that of ruxolitinib.^9,14^-^16^ This means that a large proportion of patients – very likely having a significant symptom burden^17^ – were managed with strategies that may not improve their disease control, despite the availability of more effective treatments. These patients potentially represent a cohort of unmet need.

Given that the optimal choice of management strategy varies with risk score and symptomatology, lack of prognostic information and proper recording of symptoms could result in inadequate patient management. Newer prognostic scoring tools exist for MF, such as the Mutation-Enhanced International Prognostic Score System for Transplantation-Age Patients With Primary Myelofibrosis (MIPSS70) and the Genetically Inspired Prognostic Scoring System (GIPSS), but in this study we focused on IPSS as it is the longest established and the one most commonly used during the study period. IPSS scores at diagnosis were poorly recorded in patient notes, with prognostic scoring data unavailable in 14.5% of patients. It is possible that prognostic scores were calculated but not routinely recorded; it is also possible that the information was recorded but not available to the external study team. In a similar way, symptoms were in general poorly documented despite good evidence that the majority of patients with MF have a significant symptom burden.^18^ Moreover, patient reported outcome tools such as the MPN-SAF TSS were rarely implemented in spite of extensive literature on their usefulness.^17,19^ Proper documentation of symptoms and score should always be carried out in order to streamline therapy and provide the best quality of care for patients.

Patient demographics and disease characteristics reported in this study are consistent with those previously reported for MF patients in the United Kingdom;^2,11^ clinical characteristics are also in agreement with the 2016 revision of the WHO classification of MPNs.^20^ Although anemia was documented in nearly half of all patients in this study, supportive care measures for anemia as recommended by national guidelines^21^ were infrequent, suggesting they are probably underutilized. Mortality was significant, with 47 deaths recorded during the study observation period, most of which were disease-related.

During the 2013–2018 period, clinicians in the United Kingdom applied a broad range of management strategies for the treatment of patients with MF; data also showed that some patients were managed on more than one strategy at any given time. The most common active treatments chosen as first management strategies were hydroxycarbamide and ruxolitinib, while the most common first management strategy was ‘watch and wait’ (53.5% of patients). The ‘watch and wait’ term is not universally agreed upon, with some considering it as no treatment and others considering it as treatment of some symptoms only. For this study, we defined ‘watch and wait’ as a period of time in patient management where patients were not receiving active treatment for MF.

Allogeneic HSCT was only used as a management strategy in a small proportion of patients in real-world practice. This is likely due to the fact that morbidity and mortality are particularly high in patients over 60 years old, which constitute the majority of the MF population as reported in the literature (median age 67 years)^1^ and in our study (median age 69.7 years). However, our results show that allogeneic HSCT was rarely used even in younger patients with intermediate-2 or high-risk disease. The low numbers of patients receiving allogeneic HSCT suggest that this strategy may be under-used, although other factors such as median age or transfer to a tertiary center for transplant could also have played a part.

The choice of management strategy varied according to the year of diagnosis, with a trend showing a decreased time to active treatment initiation from 2013 to 2017. This could reflect an increased awareness and uptake of existing guidelines for MF treatment, as well as an increased availability of novel therapies. Further research, taking into consideration other contributing factors (e.g. prognostic risk scores, patient demographics and choice of first line treatments), will be needed to confirm these findings.

The median time to initiation of first active treatment decreased with increasing prognostic risk score, showing that patients with higher scores were offered (or were eligible for) active treatment earlier; these observations are in agreement with current treatment recommendations for patients with intermediate to high-risk MF prognostic scores.^21,22^

Regardless of the relatively long median treatment duration with both hydroxycarbamide and ruxolitinib, a large proportion of patients discontinued treatment with these drugs. A better understanding of why patients stop treatment is needed, as well as clear guidance on when to discontinue. New therapies for MF with greater effect on disease progression and better safety profiles are also needed.

As with all retrospective studies based on secondary use of data, the accuracy and completeness of the study data was reliant on the quality of the clinical records. Recording of transitions from PV/ET to MF may be inaccurate due to non-homogeneously defined criteria.^23^ Standardization of clinical data may also be affected due to the existence of multiple sources of information. Missing data on prognostic risk scoring and changes in clinical parameters (such as spleen size and bone marrow fibrosis grades) posed a challenge for the analysis of treatment and management strategies. However a strength of this data is that it describes the clinical situation into which new care approaches or medicines need to integrate.

In summary, this is the first study in the United Kingdom to document the characteristics of patients with MF who are managed with a ‘watch and wait’ strategy in the real-world setting. The results from this observational study will help to inform the design of future studies evaluating treatment effectiveness and the benefits of earlier treatment initiation in patients with MF.

## Supplemental Material

sj-docx-1-tah-10.1177_20406207221084487 – Supplemental material for A retrospective real-world study of the current treatment pathways for myelofibrosis in the United Kingdom: the REALISM UK study Click here for additional data file. Supplemental material, sj-docx-1-tah-10.1177_20406207221084487 for A retrospective real-world study of the current treatment pathways for myelofibrosis in the United Kingdom: the REALISM UK study by Adam J. Mead, Nauman M. Butt, Waseem Nagi, Alastair Whiteway, Suriya Kirkpatrick, Ciro Rinaldi, Catherine Roughley, Sam Ackroyd, Joanne Ewing, Pratap Neelakantan, Mamta Garg, David Tucker, John Murphy, Hitesh Patel, Rozinder Bains, Gavin Chiu, Joe Hickey, Claire Harrison and Tim C. P. Somervaille in Therapeutic Advances in Hematology

sj-docx-2-tah-10.1177_20406207221084487 – Supplemental material for A retrospective real-world study of the current treatment pathways for myelofibrosis in the United Kingdom: the REALISM UK study Click here for additional data file. Supplemental material, sj-docx-2-tah-10.1177_20406207221084487 for A retrospective real-world study of the current treatment pathways for myelofibrosis in the United Kingdom: the REALISM UK study by Adam J. Mead, Nauman M. Butt, Waseem Nagi, Alastair Whiteway, Suriya Kirkpatrick, Ciro Rinaldi, Catherine Roughley, Sam Ackroyd, Joanne Ewing, Pratap Neelakantan, Mamta Garg, David Tucker, John Murphy, Hitesh Patel, Rozinder Bains, Gavin Chiu, Joe Hickey, Claire Harrison and Tim C. P. Somervaille in Therapeutic Advances in Hematology

sj-docx-3-tah-10.1177_20406207221084487 – Supplemental material for A retrospective real-world study of the current treatment pathways for myelofibrosis in the United Kingdom: the REALISM UK study Click here for additional data file. Supplemental material, sj-docx-3-tah-10.1177_20406207221084487 for A retrospective real-world study of the current treatment pathways for myelofibrosis in the United Kingdom: the REALISM UK study by Adam J. Mead, Nauman M. Butt, Waseem Nagi, Alastair Whiteway, Suriya Kirkpatrick, Ciro Rinaldi, Catherine Roughley, Sam Ackroyd, Joanne Ewing, Pratap Neelakantan, Mamta Garg, David Tucker, John Murphy, Hitesh Patel, Rozinder Bains, Gavin Chiu, Joe Hickey, Claire Harrison and Tim C. P. Somervaille in Therapeutic Advances in Hematology

sj-docx-4-tah-10.1177_20406207221084487 – Supplemental material for A retrospective real-world study of the current treatment pathways for myelofibrosis in the United Kingdom: the REALISM UK study Click here for additional data file. Supplemental material, sj-docx-4-tah-10.1177_20406207221084487 for A retrospective real-world study of the current treatment pathways for myelofibrosis in the United Kingdom: the REALISM UK study by Adam J. Mead, Nauman M. Butt, Waseem Nagi, Alastair Whiteway, Suriya Kirkpatrick, Ciro Rinaldi, Catherine Roughley, Sam Ackroyd, Joanne Ewing, Pratap Neelakantan, Mamta Garg, David Tucker, John Murphy, Hitesh Patel, Rozinder Bains, Gavin Chiu, Joe Hickey, Claire Harrison and Tim C. P. Somervaille in Therapeutic Advances in Hematology

sj-tiff-5-tah-10.1177_20406207221084487 – Supplemental material for A retrospective real-world study of the current treatment pathways for myelofibrosis in the United Kingdom: the REALISM UK study Click here for additional data file. Supplemental material, sj-tiff-5-tah-10.1177_20406207221084487 for A retrospective real-world study of the current treatment pathways for myelofibrosis in the United Kingdom: the REALISM UK study by Adam J. Mead, Nauman M. Butt, Waseem Nagi, Alastair Whiteway, Suriya Kirkpatrick, Ciro Rinaldi, Catherine Roughley, Sam Ackroyd, Joanne Ewing, Pratap Neelakantan, Mamta Garg, David Tucker, John Murphy, Hitesh Patel, Rozinder Bains, Gavin Chiu, Joe Hickey, Claire Harrison and Tim C. P. Somervaille in Therapeutic Advances in Hematology
