# Supplementary material for: A retrospective real-world study of the current treatment pathways for myelofibrosis in the United Kingdom: the REALISM UK study
Source: Ther Adv Hematol. 2022 Mar 28;13:20406207221084487. doi: 10.1177/20406207221084487 (PMC8966129; doi:10.1177/20406207221084487)
Supplement: sj-docx-1-tah-10.1177_20406207221084487 – Supplemental material for A retrospective real-world study of the current treatment pathways for myelofibrosis in the United Kingdom: the REALISM UK study [file sj-docx-1-tah-10.1177_20406207221084487.docx]

**Supplementary Table 1.** Distribution of prophylactic therapies for infection in patients treated with ruxolitinib.

| **Prophylactic therapies for infection**  **(Patients on ruxolitinib)** | | **n (therapies)** | **% (n = 16)** |
| --- | --- | --- | --- |
| Acyclovir |  | 10 | 62.5% |
| Fluoroquinolones ^a^ |  | 3 | 18.8% |
| Voriconazole |  | 2 | 12.5% |
| Lamivudine |  | 1 | 6.2% |

^a^ Ciprofloxacin or levofloxacin.
