# Supplementary material for: A retrospective real-world study of the current treatment pathways for myelofibrosis in the United Kingdom: the REALISM UK study
Source: Ther Adv Hematol. 2022 Mar 28;13:20406207221084487. doi: 10.1177/20406207221084487 (PMC8966129; doi:10.1177/20406207221084487)
Supplement: sj-docx-2-tah-10.1177_20406207221084487 – Supplemental material for A retrospective real-world study of the current treatment pathways for myelofibrosis in the United Kingdom: the REALISM UK study [file sj-docx-2-tah-10.1177_20406207221084487.docx]

**Supplementary Table 2.** Distribution of management strategies over study observation period.

| **Management strategy** | **n (patients)** | **% (n = 200)** |
| --- | --- | --- |
| AML treatment ^a^ | 1 | 1% |
| Anagrelide | 3 | 2% |
| Allogeneic HSCT | 11 | 6% |
| Allogeneic HSCT follow-up ^a^ | 10 | 5% |
| Allogeneic HSCT referral ^a^ | 2 | 1% |
| Azacitidine | 1 | 1% |
| Busulphan | 1 | 1% |
| JAK-I (as part of clinical trial) | 8 | 4% |
| Cytarabine | 1 | 1% |
| Hydroxycarbamide | 68 | 34% |
| Hydroxycarbamide + anagrelide | 4 | 2% |
| Hydroxycarbamide + azacitidine | 1 | 1% |
| Hydroxycarbamide + interferon-α | 2 | 1% |
| Interferon-α | 10 | 5% |
| Interferon-α + anagrelide | 1 | 1% |
| Radioactive phosphorous | 1 | 1% |
| Ruxolitinib | 111 | 56% |
| Ruxolitinib + anagrelide | 3 | 2% |
| Ruxolitinib + azacitidine | 1 | 1% |
| Ruxolitinib + cytarabine | 1 | 1% |
| Ruxolitinib + hydroxycarbamide | 9 | 5% |
| Ruxolitinib + thalidomide | 1 | 1% |
| Thalidomide | 3 | 2% |
| Watch and wait | 134 | 67% |

^a^ Described as such in patient records.

AML, acute myeloid leukemia; HSCT, hematopoietic stem cell transplantation; JAK-I, Janus Kinase inhibitor.

.
