# Supplementary material for: A retrospective real-world study of the current treatment pathways for myelofibrosis in the United Kingdom: the REALISM UK study
Source: Ther Adv Hematol. 2022 Mar 28;13:20406207221084487. doi: 10.1177/20406207221084487 (PMC8966129; doi:10.1177/20406207221084487)
Supplement: sj-docx-3-tah-10.1177_20406207221084487 – Supplemental material for A retrospective real-world study of the current treatment pathways for myelofibrosis in the United Kingdom: the REALISM UK study [file sj-docx-3-tah-10.1177_20406207221084487.docx]

**Supplementary Table 3.** First management strategy according to year of treatment initiation.

| **First management strategy by year initiated** | **n (patients)** | | | | | |
| --- | --- | --- | --- | --- | --- | --- |
|  | **2013** | **2014** | **2015** | **2016** | **2017** | **2018** |
| Watch and wait | 11 (10%) | 26 (24%) | 24 (22%) | 26 (24%) | 20 (19%) | 0 (0%) |
| Ruxolitinib | 2 (6%) | 8 (23%) | 8 (23%) | 6 (17%) | 11 (31%) | 0 (0%) |
| Hydroxycarbamide | 5 (11%) | 9 (20%) | 9 (20%) | 14 (31%) | 7 (16%) | 1 (2%) |
| Interferon-α | 0 (0%) | 0 (0%) | 1 (25%) | 2 (50%) | 1 (25%) | 0 (0%) |
| Anagrelide | 0 (0%) | 0 (0%) | 1 (33%) | 1 (33%) | 1 (33%) | 0 (0%) |
| JAK-I (as part of clinical trial) | 1 (50%) | 0 (0%) | 1 (50%) | 0 (0%) | 0 (0%) | 0 (0%) |
| Hydroxycarbamide + anagrelide | 0 (0%) | 0 (0%) | 1 (33%) | 1 (33%) | 1 (33%) | 0 (0%) |
| Ruxolitinib + anagrelide | 0 (0%) | 0 (0%) | 1 (100%) | 0 (0%) | 0 (0%) | 0 (0%) |

JAK-I, Janus Kinase inhibitor.
