# Supplementary material for: A retrospective real-world study of the current treatment pathways for myelofibrosis in the United Kingdom: the REALISM UK study
Source: Ther Adv Hematol. 2022 Mar 28;13:20406207221084487. doi: 10.1177/20406207221084487 (PMC8966129; doi:10.1177/20406207221084487)
Supplement: sj-docx-4-tah-10.1177_20406207221084487 – Supplemental material for A retrospective real-world study of the current treatment pathways for myelofibrosis in the United Kingdom: the REALISM UK study [file sj-docx-4-tah-10.1177_20406207221084487.docx]

**Supplementary Table 4.** Time to initiation of first active treatment by year of diagnosis and IPSS group.

| **Time to first active treatment (days) stratified by year of diagnosis and IPSS score at diagnosis** | | **n (patients)** | **Mean** | **SD** | **Median** | **IQR** | **Range** |
| --- | --- | --- | --- | --- | --- | --- | --- |
| **2013** | Low (0) | 2 | 0.0 | 0.0 | 0.0 | 0.0 to 0.0 | 0.0 to 0.0 |
|  | Intermediate - 1 | 9 | 335.4 | 567.5 | 152.0 | 0.0 to 239.0 | 0.0 to 1755.0 |
|  | Intermediate - 2 | 6 | 487.3 | 562.0 | 385.0 | 29.8 to 707.3 | 0.0 to 1428.0 |
|  | High >=3 | 3 | 303.0 | 524.8 | 0.0 | 0.0 to 454.5 | 0.0 to 909.0 |
| **2014** | Low (0) | 8 | 190.6 | 265.2 | 76.5 | 0.0 to 263.8 | 0.0 to 647.0 |
|  | Intermediate - 1 | 14 | 587.1 | 550.6 | 415.5 | 107.8 to 1127.3 | 0.0 to 1463.0 |
|  | Intermediate - 2 | 14 | 167.9 | 262.4 | 0.0 | 0.0 to 246.3 | 0.0 to 903.0 |
|  | High >=3 | 7 | 212.9 | 311.7 | 61.0 | 0.0 to 289.0 | 0.0 to 851.0 |
| **2015** | Low (0) | 6 | 692.5 | 545.9 | 970.5 | 242.3 to 977.3 | 0.0 to 1235.0 |
|  | Intermediate - 1 | 18 | 312.9 | 346.8 | 200.5 | 0.0 to 537.0 | 0.0 to 953.0 |
|  | Intermediate - 2 | 14 | 134.9 | 260.6 | 0.0 | 0.0 to 81.3 | 0.0 to 869.0 |
|  | High >=3 | 8 | 184.9 | 240.1 | 70.0 | 0.0 to 315.3 | 0.0 to 610.0 |
| **2016** | Low (0) | 7 | 331.6 | 308.2 | 376.0 | 51.0 to 555.5 | 0.0 to 732.0 |
|  | Intermediate - 1 | 14 | 141.3 | 209.0 | 31.0 | 0.0 to 260.3 | 0.0 to 591.0 |
|  | Intermediate - 2 | 17 | 185.9 | 242.6 | 58.0 | 0.0 to 319.0 | 0.0 to 635.0 |
|  | High >=3 | 12 | 56.8 | 120.1 | 0.0 | 0.0 to 45.5 | 0.0 to 394.0 |
| **2017** | Low (0) | 3 | 137.3 | 146.8 | 120.0 | 60.0 to 206.0 | 0.0 to 292.0 |
|  | Intermediate - 1 | 14 | 135.0 | 185.8 | 31.5 | 0.0 to 246.5 | 0.0 to 499.0 |
|  | Intermediate - 2 | 14 | 86.7 | 164.7 | 0.0 | 0.0 to 51.8 | 0.0 to 490.0 |
|  | High >=3 | 9 | 91.2 | 130.9 | 21.0 | 0.0 to 192.0 | 0.0 to 343.0 |
| **2018** | Low (0) | 0 |  |  |  |  |  |
|  | Intermediate - 1 | 1 | 0.0 | - | 0.0 | 0.0 to 0.0 | 0.0 to 0.0 |
|  | Intermediate - 2 | 0 |  |  |  |  |  |
|  | High >=3 | 0 |  |  |  |  |  |

IPSS, International Prognostic Scoring System; IQR, interquartile range; SD, standard deviation.
