# Supplementary figures and images for: A retrospective real-world study of the current treatment pathways for myelofibrosis in the United Kingdom: the REALISM UK study
Source: Ther Adv Hematol. 2022 Mar 28;13:20406207221084487. doi: 10.1177/20406207221084487 (PMC8966129; doi:10.1177/20406207221084487)

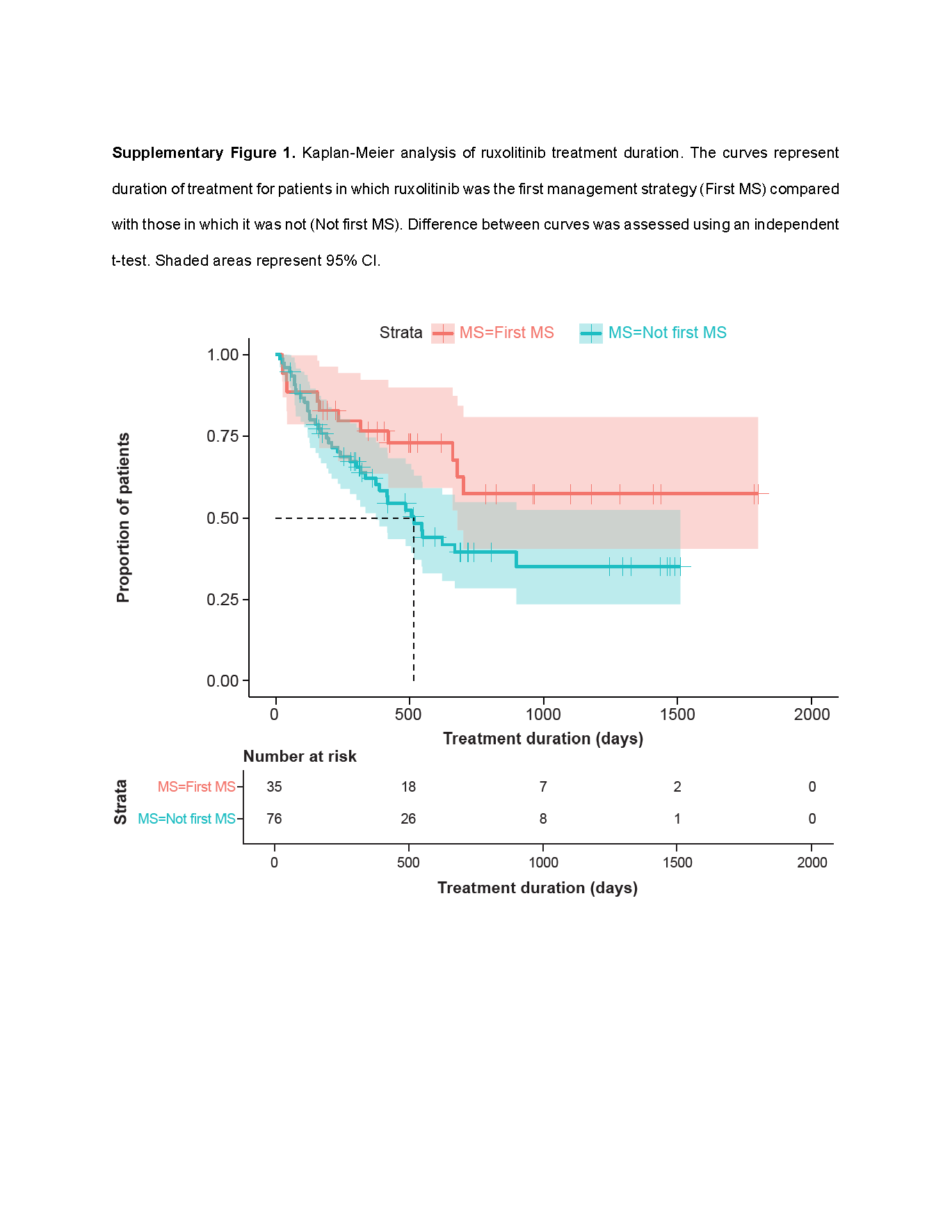

Supplement: sj-tiff-5-tah-10.1177_20406207221084487 – Supplemental material for A retrospective real-world study of the current treatment pathways for myelofibrosis in the United Kingdom: the REALISM UK study [file sj-tiff-5-tah-10.1177_20406207221084487.tiff]
